# DNA Methylation Score as a Biomarker in Newborns for Sustained Maternal Smoking during Pregnancy

**DOI:** 10.1289/EHP333

**Published:** 2016-06-21

**Authors:** Sarah E. Reese, Shanshan Zhao, Michael C. Wu, Bonnie R. Joubert, Christine L. Parr, Siri E. Håberg, Per Magne Ueland, Roy M. Nilsen, Øivind Midttun, Stein Emil Vollset, Shyamal D. Peddada, Wenche Nystad, Stephanie J. London

**Affiliations:** 1 Epidemiology Branch, and; 2 Biostatistics and Computational Biology Branch, National Institute of Environmental Health Sciences (NIEHS), National Institutes of Health (NIH), Department of Health and Human Services (DHHS), Research Triangle Park, North Carolina, USA; 3 Public Health Sciences Division, Fred Hutchinson Cancer Research Center, Seattle, Washington, USA; 4 Population Health Branch, NIEHS, NIH, DHHS, Research Triangle Park, North Carolina, USA; 5 Department of Chronic Diseases, and; 6 Department of Management and Staff, Norwegian Institute of Public Health, Oslo, Norway; 7 Department of Clinical Science, University of Bergen, Bergen, Norway; 8 Laboratory of Clinical Biochemistry, Haukeland University Hospital, Bergen, Norway; 9 Department of Global Public Health and Primary Care, University of Bergen, Bergen, Norway; 10 Center for Clinical Research, Haukeland University Hospital, Bergen, Norway; 11 Bevital A/S, Bergen, Norway; 12 Center for Disease Burden, Norwegian Institute of Public Health, Oslo/Bergen, Norway

## Abstract

**Background:**

Maternal smoking during pregnancy, especially when sustained, leads to numerous adverse health outcomes in offspring. Pregnant women disproportionately underreport smoking and smokers tend to have lower follow-up rates to repeat questionnaires. Missing, incomplete, or inaccurate data on presence and duration of smoking in pregnancy impairs identification of novel health effects and limits adjustment for smoking in studies of other pregnancy exposures. An objective biomarker in newborns of maternal smoking during pregnancy would be valuable.

**Objectives:**

We developed a biomarker of sustained maternal smoking in pregnancy using common DNA methylation platforms.

**Methods:**

Using a dimension reduction method, we developed and tested a numeric score in newborns to reflect sustained maternal smoking in pregnancy from data on cotinine, a short-term smoking biomarker measured mid-pregnancy, and Illumina450K cord blood DNA methylation from newborns in the Norwegian Mother and Child Cohort Study (MoBa).

**Results:**

This score reliably predicted smoking status in the training set (*n* = 1,057; accuracy = 96%, sensitivity = 80%, specificity = 98%). Sensitivity (58%) was predictably lower in the much smaller test set (*n* = 221), but accuracy (91%) and specificity (97%) remained high. Reduced birth weight, a well-known effect of maternal smoking, was as strongly related to the score as to cotinine. A three-site score had lower, but acceptable, performance (accuracy_train_ = 82%, accuracy_test_ = 83%).

**Conclusions:**

Our smoking methylation score represents a promising novel biomarker of sustained maternal smoking during pregnancy easily calculated with Illumina450K or IlluminaEPIC data. It may help identify novel health impacts and improve adjustment for smoking when studying other risk factors with more subtle effects.

**Citation:**

Reese SE, Zhao S, Wu MC, Joubert BR, Parr CL, Håberg SE, Ueland PM, Nilsen RM, Midttun Ø, Vollset SE, Peddada SD, Nystad W, London SJ. 2017. DNA methylation score as a biomarker in newborns for sustained maternal smoking during pregnancy. Environ Health Perspect 125:760–766; http://dx.doi.org/10.1289/EHP333

## Introduction

Despite years of health warnings and cessation campaigns, smoking during pregnancy remains an important public health problem (Murin et al. 2011). Women who smoke during pregnancy are more likely to have children with lower birth weight, preterm delivery, reduced lung function, asthma, attention deficit hyperactivity disorder (ADHD), orofacial clefts, and other malformations (DHHS 2014). Emerging evidence links additional health outcomes in children to maternal smoking (Mund et al. 2013). Because of the consistent and important effects of maternal smoking on child health, it is crucial to carefully adjust for smoking when investigating effects of other *in utero* environmental exposures that may have more subtle effects.

Various newborn adverse health outcomes related to maternal smoking, including reduced birth weight, have been shown to be mitigated by cessation (DHHS 2014), suggesting that sustained smoking during pregnancy rather than simply any smoking during pregnancy is the important parameter to assess in epidemiologic studies. Using a genome-wide platform [Illumina® Infinium HumanMethylation450 BeadChip (Illumina®450K)], Joubert et al. (2012) reported that maternal smoking during pregnancy was associated with differential DNA methylation in newborns at specific loci that replicated in a second population. Subsequent reports have consistently confirmed and extended these findings (Joubert et al. 2016). Joubert et al. (2014) subsequently reported that the DNA methylation signals observed in newborns reflected sustained smoking, defined by cotinine measured at about 18 weeks of gestation, rather than transient smoking; these signals were not seen when women quit smoking earlier in pregnancy.

Smoking during pregnancy is generally assessed in epidemiologic studies by questionnaires. Studies vary in the number of time points at which smoking information is collected and, even when complete histories across pregnancy are sought, missing questionnaire data at one or more time points decreases sample size for assessment of sustained smoking. Smokers tend to have lower response rates to follow-up questionnaires (Jacobsen and Thelle 1988). While a positive self-report of smoking is reliable, pregnant women are more likely to underreport smoking than are women of the same age who are not pregnant (Dietz et al. 2011; Kvalvik et al. 2012), likely due to the well-known negative effects of this exposure on the child. Cotinine is the best biomarker of smoking status available (Benowitz 1996; Kvalvik et al. 2012), but it has a half-life of only 17 hr in nonpregnant women (Benowitz 1996) and 9 hr in pregnant women (Dempsey et al. 2002). There have been recent attempts to develop biomarkers of long-term smoking exposure in adults using the Illumina®450K platform (Shenker et al. 2013; Zhang et al. 2016). However, this has not been done in newborns to reflect exposure to maternal smoking during pregnancy.

The goal of this paper is to develop, using the Illumina®450K methylation platform, a biomarker in newborns of sustained smoking by the mother during pregnancy that can be easily applied to other newborn studies with either Illumina®450K or Illumina® Infinium® MethylationEPIC BeadChip (Illumina®EPIC) methylation data. A biomarker of this nature will be useful in studies of childhood health outcomes to fill in the inevitable missing data on whether or for how long a mother smoked, when limited data were collected on timing of smoking, and to validate self-reports of nonsmoking. While statistical methods exist to fill in missing data, such as multiple imputation, these are inferior to a direct and objective biomarker. Further, these methods involve assumptions about the random nature of the missing data (Sterne et al. 2009) that are unlikely to hold for smoking, especially during pregnancy. We used two existing data sets with Illumina®450K methylation measured in newborns and cotinine measured in maternal plasma during pregnancy to develop and test a methylation score to predict smoking. We also examined the association between the resulting methylation score and reduced birth weight, a well established consequence of maternal smoking during pregnancy (DHHS 2014).

## Methods

### Study Population

The Norwegian Mother and Child Cohort Study (MoBa) is a large population-based pregnancy study conducted by the Norwegian Institute of Public Health targeting all women who gave birth in Norway from 1999 to 2008 (Magnus et al. 2006; Rønningen et al. 2006). Blood samples were obtained from the mother during pregnancy and from newborns (cord blood). Here we analyzed a subcohort of MoBa participants (born 2002–2004) with Illumina®450K methylation data measured from newborn DNA and cotinine measured from maternal plasma at about gestational week 18 of pregnancy (Joubert et al. 2012). The Illumina®450K methylation data were generated in two different analytic batches: MoBa1 (*n* = 1,068, generated in 2011) and MoBa2 (*n* = 222, generated in 2013). We used the first data set (MoBa1) analyzed by Joubert et al. (2012), as our training set. The second data set (MoBa2) served as our test data set.

Exposure to nicotine from sources other than cigarette smoking could be reflected in cotinine levels but are not expected to generate the same methylation signals (Besingi and Johansson 2014); therefore, we excluded the 10 subjects from the training set and one subject from the test set who reported use during pregnancy of snuff/chewing tobacco, nicotine gum, nicotine patch, or nicotine inhaler. One additional subject was excluded from the training set due to missing cotinine data. This left us with 1,057 subjects in the training set and 221 in the test set for analyses.

The MoBa study has been approved by the Regional Committee for Ethics in Medical Research, the Norwegian Data Inspectorate, and the Institutional Review Board of the National Institute of Environmental Health Sciences. Written informed consent was obtained from all participants.

### Laboratory Measurements

***Cotinine.*** Cotinine concentrations were measured in maternal plasma collected at approximately 18 weeks gestation (Kvalvik et al. 2012) using liquid chromatography-tandem mass spectrometry at BEVITAL AS (http://www.bevital.no) (Midttun et al. 2009).

***Methylation data.*** We measured DNA methylation in cord blood samples at 485,577 CpG sites using the Illumina®450K (Illumina, Inc., San Diego, CA) (Bibikova et al. 2011; Sandoval et al. 2011). Bisulfite conversion was performed using the EZ-96 DNA Methylation™ kit (Zymo Research Corporation, Irvine, CA). All quality control and data processing was done as described previously (Joubert et al. 2012). Briefly, samples were omitted if the average detection *p*-value across all probes was < 0.05 or they were labeled as failed by the laboratory, they were identified as a gender outlier, or they were a blind duplicate of another sample included in the data set. CpGs that were missing chromosome data, were missing more than 10% of data across samples, or were on chromosome X or Y were omitted. Joubert et al. (2012) found no evidence of batch effects in these data, which were generated over < 4 weeks. Beta values, β, were calculated using the GenomeStudio methylation software (version 1.0; Illumina® Inc.) as the ratio of the intensity of the methylated allele to the sum of the intensities of the methylated and unmethylated alleles plus a constant. The beta values were additionally logit transformed to obtain the log ratio,


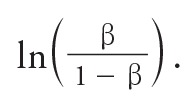
.

### Definition of Sustained Smoking in this Analysis

We used the term sustained smoking as defined in our previous report (Joubert et al. 2014) where we found that the methylation signals were observed in newborns with mothers in this group but not in mothers who quit early in pregnancy. Among the 1,278 pregnancies across the training and test sets, we examined the timing of quitting smoking during pregnancy using questionnaire data collected at two time points in pregnancy (approximately weeks 17 and 30 of gestation). Among these women, 127 reported smoking at the beginning of pregnancy and did not report quitting. Among the 253 who reported quitting during pregnancy, there were 54 who did not report in which week of pregnancy they quit, 184 who reported quitting by 18 weeks, and 15 who reported quitting after 18 weeks. Thus the vast majority of women who reported that they stopped smoking during pregnancy did so by 18 weeks. Our cotinine value measured at about 18 weeks identifies women who were still smoking at this time point. When considered in combination with our questionnaire data, a cotinine value in the active smoking range reflects smoking into the second trimester, as opposed to smoking that stopped early in pregnancy; and, for the vast majority of women who smoked at the onset of pregnancy, the value correlates with smoking through most of pregnancy. We therefore refer to smoking detected by cotinine > 56.8 nmol/L (Shaw et al. 2009) at about 18 weeks or self-reported later in pregnancy (17 or 30 weeks) as sustained smoking during pregnancy in this analysis.

***Cotinine-based classification of sustained smoking during pregnancy.*** We refer to the smoking variable based solely on cotinine dichotomized at 56.8 nmol/L as cotinine-based sustained smoking.

***Self-report based classification of sustained smoking during pregnancy.*** The self-reported sustained smoking variable was created from data from two questionnaires, one administered at about 17 weeks of pregnancy and one administered at about 30 weeks, supplemented with information collected from mothers at birth from the Medical Birth Registry of Norway (MBRN). This variable classifies mothers who reported that they were sometimes or daily smokers as smokers, and mothers who reported that they never smoked, quit before pregnancy, or stopped smoking early in pregnancy as nonsmokers.

***Combined classification of sustained smoking during pregnancy.*** We also created a combined sustained smoking variable that classifies mothers based on cotinine levels above 56.8 nmol/L as smokers combined with mothers who self-reported as daily smokers whether or not their cotinine value exceeded this threshold. This combined sustained smoking variable reclassified 10 individuals in the training set and 1 person in the test set as smokers who had been classified as nonsmokers according to the cotinine-based sustained smoking variable.

## Statistical Methods

### Development of Smoking Biomarker on Training Data

We performed a genome-wide robust linear regression (Fox and Weisberg 2011) on the training set (MoBa1) using the combined sustained smoking variable as the dichotomous predictor and the log ratios of the DNA methylation data as the response variable. These were non-normalized as in Joubert et al. (2012) so as to closely replicate these results. The top 200 most significant CpGs were selected, consistent with the sure independent screening approach suggested by Fan and Lv (2008). We then cross-referenced the 200 CpGs with lists of potentially problematic probes (Chen et al. 2013), including those that have single nucleotide polymorphisms nearby. We visually inspected the distributions of all CpGs that overlapped with these lists and removed 5 CpGs with nonunimodal distributions from further analysis. The remaining 195 CpGs were used in the logistic least absolute shrinkage and selection operator (LASSO) model to choose a set of CpGs for use in the calculation of the smoking score (Hastie et al. 2009; Tibshirani 1996).

We used the untransformed methylation beta values as the predictors of maternal smoking because it has become more common to analyze Illumina®450K data on the natural scale. In previous studies, results of classification methods were not significantly different when using beta values versus log ratios for large sample sizes (Zhuang et al. 2012). To account for the randomness of the LASSO procedure (Hastie et al. 2009; Tibshirani 1996), we performed it 100 times. After running the 100 iterations, we selected the subset of CpGs that appeared in all 100 to choose a robust subset of CpGs that might be more applicable to other studies. A smoking score was then calculated as the linear combination of the subset of CpGs and the logistic LASSO regression coefficients.

Receiver operating characteristic (ROC) analysis [version 1.8 (pROC); R Development Core Team] (Metz 1978) was used to establish a threshold, based on the logistic LASSO regression coefficients [version 2.0-5 (glmnet); R Development Core Team], for the smoking methylation score to classify newborns according to exposure to a mother with sustained smoking during pregnancy using the combined variable described above. We set the threshold to minimize the sum of false positives and false negatives with the restriction that the sensitivity had to be at least 80%. False positives are subjects misclassified as offspring of smoking mothers, whose mothers did not smoke according to their combined self-report and cotinine measurements. False negatives are subjects misclassified as offspring of mothers who did not smoke, who appear to be smokers based on their combined self-report and cotinine values. We calculated the area under the curve (AUC) and used the threshold to classify samples and to calculate the accuracy, sensitivity, and specificity.

### Validation of Smoking Biomarker on Test Data

Using the same logistic LASSO regression coefficients and threshold value, we calculated the smoking methylation score for the test set (MoBa2) and performed ROC analysis at the threshold established above to calculate the accuracy, sensitivity, and specificity.

### Comparing Different Smoking Variables to Train the Score

Several additional analyses were performed to assess how the LASSO regression results changed when using other smoking variables to train the model rather than combined sustained smoking. We focused on combined sustained smoking because, although cotinine is an objective measure and the best available biomarker of smoking, it is relatively short term. Most pregnant women in our study were not heavy smokers, and many did not smoke daily. Thus, if a woman refrained from smoking on the day of her clinic visit when blood for cotinine was drawn, the value might be in the nonsmoking range. Because pregnant women are exceedingly unlikely to claim to be smokers when they are not, it seems imprudent to overwrite a positive self-report of smoking because of a cotinine value below our cutoff. In addition to this primary smoking variable (i.e., combined sustained smoking), we trained our model using two additional smoking variables: cotinine-based sustained smoking, which was based only on the cotinine measurement, and self-reported sustained smoking, which was based only on questionnaire data.

We performed an additional sensitivity analysis using a naïve CpG selection approach that included only the three loci replicated at strict Bonferroni significance in Joubert et al. (2012) to form the smoking methylation score. This approach used the most significant CpG from each of these three loci (*AHRR*, *GFI1*, and *CYP1A1*) from our genome-wide analysis and the corresponding robust linear regression coefficients to compute the smoking methylation score.

Illumina® recently released the EPIC BeadChip that covers more than 850K CpG sites (Moran et al. 2016). Approximately 42,000 of the Illumina®450K CpGs are not included on Illumina®EPIC. Because we do not have Illumina®EPIC data, we assessed the performance of the score trained on the Ilumina®450K data after deleting CpGs that do not overlap between the two platforms.

The AUC, accuracy, sensitivity, and specificity were used to evaluate the performance of the methylation score created in these different additional analyses.

### Birth Weight in Relation to the Different Smoking Variables

We examined how our methylation score relates to a known newborn health outcome of having a mother who smoked during pregnancy. We chose birth weight because of the well-established inverse association with maternal smoking during pregnancy (DHHS 2014). We performed a linear regression analysis to compare the association between birth weight and various smoking variables: sustained smoking based on our newly created smoking methylation score, cotinine-based sustained smoking, self-reported sustained smoking, combined sustained smoking (using both self-report and cotinine), and a self-report variable for *any* (yes or no) smoking during the pregnancy whether sustained or not. We appreciate that there is some circularity because we developed the score in the training portion of the data using the combined sustained smoking variable as the gold standard.

The birth weight variable came from the MBRN (Irgens 2000). Covariates included in all birth weight models were gender, gestational age, maternal education, maternal age, parity, and the selection variable for the data set. We also created a crude model without the smoking variable for comparison.

We assessed fit of the birth weight models using likelihood ratio tests (LRT) comparing models including a smoking variable to the crude model. We used root mean square error (RMSE) to assess how well each model estimated birth weight. The smaller the RMSE the better the model estimated birth weight.

All analyses were performed in R (version 3.2.4; R Development Core Team) using glmnet, pROC, MASS, sandwich, and lmtest.

## Results

The percentage of mothers positive for combined sustained smoking during pregnancy was similar in the training and test sets (Training: 13.0%; Test: 14.0%; *p*-value = 0.34; Table 1). Among these smokers, the amount smoked was low (median = 5 cigarettes per day) in both the training and test sets (Table 1).

**Table 1:** Descriptive statistics of sustained smoking variables, cotinine, and quantity smoked.

Variable	Category	Training (MoBa1; *n* = 1,057)	Test (MoBa2; *n *= 221)
Cotinine-based sustained smoking; *n* (%)^*a*^	No	930 (88.0)	191 (86.4)
	Yes	127 (12.0)	30 (13.6)
Self-reported sustained smoking; *n* (%)	No	936 (88.6)	191 (86.4)
	Yes	121 (11.4)	30 (13.6)
Combined sustained smoking; *n* (%)	No	920 (87.0)	190 (86.0)
	Yes	137 (13.0)	31 (14.0)
Cotinine values by sustained smoking category^*b*^ (mean ± SD)	No	0.7 ± 3.4	0.7 ± 2.2
	Yes	424 ± 337	497 ± 301
Number of cigarettes per day (among smokers^*b*^; median (IQR))		5 (2–10)	5 (3–7)
^***a***^Based on cotinine measured in maternal plasma collected at about 18 weeks of pregnancy, values > 56.8 nmol/L classified as Yes. ^***b***^Based on the combined sustained smoking variable.			

The iterative logistic LASSO AUC cross-validation procedure, a procedure to choose the CpGs most predictive of combined sustained smoking, identified 28 CpGs retained in all 100 runs in the training set (see Table S1). As expected, there was substantial overlap of the CpGs on this list and those reported by Joubert et al. (2012)—5 of the original 10 loci were identified. The distributions of the calculated smoking methylation score for the training set by levels of our combined sustained smoking variable are displayed in Figure S1A. In the ROC analysis for the training set (*n* = 1,057), the smoking methylation score compared well to the combined sustained smoking variable (AUC = 0.96 [95% confidence interval (CI): 0.95, 0.98]; see Figure S2). The resulting threshold value for the smoking methylation score was –0.37 with an accuracy of 96%, sensitivity of 80%, and specificity of 98% (Table 2 Model c). At this threshold, there were 19 (1.8%) false positives (nonsmokers who were classified as smokers) and 27 (2.6%) false negatives in the training set.

**Table 2:** Logistic LASSO results for main and additional analyses.

Model	Data set	*q*	AUC (CI)	Threshold	Accuracy (CI)	Sensitivity (CI)	Specificity (CI)	FN (%)	FP (%)
a Cotinine-based sustained smoking	Training^*a*^	24	0.97 (0.95, 0.99)	–9.09	0.95 (0.94, 0.97)	0.83 (0.76, 0.89)	0.97 (0.96, 0.98)	22 (2.1)	27 (2.6)
	Test^*a*^		0.88 (0.80, 0.96)		0.90 (0.86, 0.93)	0.63 (0.47, 0.80)	0.94 (0.90, 0.97)	11 (5.0)	12 (5.4)
b Self-reported sustained smoking	Training^*a*^	12	0.93 (0.90, 0.96)	–11.71	0.92 (0.90, 0.94)	0.81 (0.74, 0.88)	0.93 (0.92, 0.95)	23 (2.2)	64 (6.0)
	Test^*a*^		0.82 (0.74, 0.91)		0.90 (0.86, 0.93)	0.47 (0.30, 0.63)	0.96 (0.94, 0.99)	16 (7.2)	7 (3.2)
c Combined sustained smoking^*b*^	Training^*a*^	28	0.96 (0.95, 0.98)	–0.37	0.96 (0.94, 0.97)	0.80 (0.74, 0.87)	0.98 (0.97, 0.99)	27 (2.6)	19 (1.8)
	Test^*a*^		0.90 (0.83, 0.97)		0.91 (0.88, 0.95)	0.58 (0.39, 0.74)	0.97 (0.94, 0.99)	13 (5.9)	6 (2.7)
d Naïve CpG selection^*c*^	Training^*a*^	3	0.89 (0.86, 0.92)	–0.47	0.82 (0.80, 0.84)	0.81 (0.74, 0.87)	0.82 (0.80, 0.85)	24 (2.3)	166 (15.7)
	Test^*a*^		0.82 (0.73, 0.91)		0.83 (0.78, 0.88)	0.60 (0.43, 0.77)	0.87 (0.82, 0.92)	12 (5.4)	25 (11.3)
Note: The number of CpGs (q) used to calculate the smoking methylation score, area under the curve (AUC) and 95% confidence interval (CI), smoking methylation score threshold, accuracy and CI, sensitivity and CI, specificity and CI, and number and percentage of false negatives (FN) and false positives (FP). ^***a***^Training *n *= 1,057; Test *n *= 221. ^***b***^In the combined sustained smoking variable, a woman’s positive report of daily smoking during pregnancy overrides a cotinine value of ≤ 56.8 nmol/L used to classify a woman as a nonsmoker in the cotinine-based sustained smoking variable. ^***c***^Naïve CpG selection refers to the smoking score calculated using the three CpGs from the loci replicated at strict Bonferroni significance in Joubert et al. (2012). These CpGs and corresponding coefficients are cg05575921 (–0.558; *AHRR*), cg14179389 (–0.555; *GFI1*), cg18092474 (0.205; *CYP1A1*).									

For the test set (*n* = 221) the AUC was 0.90 (95% CI: 0.83, 0.97; see Figure S2), using the same regression coefficients from the LASSO to calculate the smoking methylation score (see Figure S1B) and the same threshold value for the ROC analysis. As expected, the performance of the smoking methylation score was not as high in this much smaller test set (Table 2 Model c): sensitivity was reduced to 58%, although accuracy (91%) and specificity (97%) were only slightly lower than in the training set. In the test set there were 6 (2.7%) false positives and 13 (5.9%) false negatives.

### Additional Analyses

As expected, cotinine-based sustained smoking and self-reported sustained smoking differed slightly [see Table S2; phi coefficient = 0.79 (Training set) and 0.81 (Test set)]. Therefore, we compared our main analysis (combined sustained smoking, Table 2 Model c; see also Table S1) to models where we trained the smoking methylation score using the cotinine-based sustained smoking variable (Table 2 Model a; see also Table S3) or separately, the self-reported sustained smoking variable (Table 2 Model b; see also Table S4). Table 2 shows the number of CpGs (*q*) used to calculate the smoking methylation score and the results of the ROC analysis for the smoking methylation scores calculated using the three different smoking variables. The predictive ability of the smoking methylation score was best when trained on the combined sustained smoking. As expected, in all models the sensitivity in the smaller test set was substantially reduced compared with the larger training set. The specificity remained high, only slightly reduced, for the test set compared with the training set.

The naïve approach using only the three replicated CpGs does not predict smoking status as reliably as the LASSO model trained on combined sustained smoking and resulted in lower sensitivity and considerably lower specificity in both the training and test sets (Table 2 Model d) although it had acceptable performance (training set AUC = 0.89, test set AUC = 0.82).

Only 2 of the 28 CpGs identified in the combined sustained smoking score are not included in the Illumina®EPIC array (cg00709966 and cg11864574). Leaving these 2 CpGs out made very little difference in the performance of the score (see Tables S5 and S6).

### Birth Weight Analysis

Using linear regression models, we compared the association between birth weight and smoking, classified variously as exposed based on the smoking methylation score (12.7% prevalence), cotinine-based sustained smoking (12.2%), self-reported sustained smoking (11.6%), combined sustained smoking (13.2%), and an additional variable for self-report of any smoking during pregnancy whether sustained or not (yes versus no; yes = 28.3%; see Table S7). Tables S7 and S8 give descriptive statistics in the training data for the smoking variables and covariates included in the models. Table 3 shows the resulting coefficients and standard errors from the linear regression models, the Akaike information criterion (AIC), log-likelihood, and *p*-value resulting from the likelihood ratio test to the crude model. The RMSE did not distinguish much between models (range 444.06–445.62). This is not surprising given that maternal smoking leads only to a modest decrement in birth weight, and thus, is not its major determinant; in these data the maximum percent of variation explained was 33.2% (range 32.6–33.2%). Although the differences were miniscule, the sustained smoking models all performed significantly better than the crude model (Table 3) whereas the any smoking variable did not perform better than the crude model.

**Table 3:** Birth weight regression analysis results on the training data (*n *= 1,039).

Model	Coefficient	SE	P_LM_^*a*^	AIC	logL	P_LRT_	RMSE
Crude	NA	NA	NA	15645.5	–7812.8	NA	446.11
Methylation score class	–130.9	42.81	0.0023	15638.1	–7808.1	0.0022	444.10
Cotinine-based sustained smoking	–133.3	44.08	0.0026	15638.3	–7808.2	0.0024	444.14
Self-reported sustained smoking	–120.4	44.95	0.0075	15640.3	–7809.2	0.0072	444.56
Combined sustained smoking^*b*^	–131.4	42.62	0.0021	15638.0	–7808.0	0.0020	444.06
Self-reported any smoking	–48.2	32.18	0.1348	15645.3	–7811.6	0.1329	445.62
Note: Coefficient, standard error (SE), and linear model *p*-value (P_LM_) for each model with a smoking variable, and Akaike information criterion (AIC), log likelihood (logL), likelihood-ratio test to the crude model, *p*-value (P_LRT_), and root mean squared error (RMSE) for each model. All models adjusted for gender, gestational age, maternal education, maternal age, parity, and selection. ^***a***^*p*-Values < 0.05 were considered to represent statistical significance. ^***b***^In the combined sustained smoking variable, a woman’s self-report of daily smoking during pregnancy overrides a cotinine value of ≤ 56.8 nmol/L used to classify a woman as a nonsmoker in the cotinine-based sustained smoking variable.							

## Discussion

We developed a novel biomarker in newborns of sustained maternal smoking in pregnancy using methylation values in newborns from the Illumina®450K platform. This biomarker is a smoking methylation score that incorporates the subset of 28 CpGs we found to be most predictive of maternal smoking status from a logistic LASSO model. The sensitivity was high in the training set but lower, as expected, in the much smaller separate test set; however, the specificity remained high in both. When we evaluated the relationship with reduced birth weight, a well-established health effect of maternal smoking, we found that our smoking methylation biomarker performed about the same as the cotinine-based sustained smoking, self-reported sustained smoking, combined sustained smoking incorporating self-report and cotinine, and substantially better than self-report of any smoking in pregnancy.

The score that we developed is intended for studies with Illumina®450K methylation data. For studies with the new Illumina®EPIC array, the score can be directly applied using the CpGs from our score that overlap with those on the Illumina®EPIC array with little loss of performance. Our work also allows comparison with a naïve method based not on any dimension reduction method but simply on three replicated top loci from Joubert et al. (2012). Interestingly, this naïve three CpG score performed relatively well given how little epigenetic information was included (training accuracy 82% versus 96% from the LASSO). For studies without Illumina®450K or Illumina®EPIC data, this score could be implemented by assessing methylation at these three loci using pyrosequencing or other methods (Roessler and Lehmann 2015; Wani and Aldape 2016; Wiencke et al. 2014).

Previous studies have developed biomarkers of smoking in adults from methylation data. Shenker et al. (2013) developed a methylation index based on a linear combination of methylation values of four CpGs and the coefficients from their genome-wide analysis. Zhang et al. (2016) developed a biomarker based on two CpGs that were strongly associated with all-cause, cardiovascular, and cancer mortality. Philibert et al. (2015) investigated the use of five CpGs as potential indicators of smoking for use in clinical settings. We developed a biomarker of sustained smoking in pregnancy using genome-wide data, which retained a larger number of CpGs (*q* = 28). While there are several dimension reduction methods to choose from, we chose LASSO because it generally selects a more parsimonious set of features and it is difficult to show a significant difference in performance between the methods (Hastie et al. 2009). This smaller set of CpGs expected to be selected by the LASSO allows the smoking methylation score to be more easily implemented in other studies.

Recent studies have shown that many of the smoking methylation signals seen in newborns persist into childhood. For example, the three CpGs in our naïve score are also related to sustained maternal smoking during pregnancy in several studies of older children, but the effects are attenuated with the passage of time (Küpers et al. 2015; Ladd-Acosta et al. 2016; Lee et al. 2015; Richmond et al. 2015).

The smoking methylation score provides studies that lack cotinine values or have incomplete self-reported smoking histories with an easy to calculate, objective biomarker in newborns of having a mother who smoked during most of the pregnancy as well as a validation of self-reported nonsmoking. It can be used to fill in missing data on smoking or its timing throughout pregnancy. A biomarker is superior to statistical methods to fill in missing data, such as multiple imputation. Our score is simple to compute in other newborn data sets with Illumina®450K or Illumina®EPIC methylation data to generate a biomarker in newborns of sustained smoking in pregnancy. The score is a simple linear combination of the methylation values of 28 CpGs and a vector of logistic LASSO regression coefficients, which we have provided in Table S1. It is known that positive self-reports of smoking are reliable but that some smokers may falsely deny smoking. Because of the well-publicized adverse effects of smoking during pregnancy on offspring, pregnant smokers are more likely to deny smoking than are other smokers of reproductive age who are not pregnant (Dietz et al. 2011; Kvalvik et al. 2012). Thus, in studying effects of maternal smoking in pregnancy on health outcomes in children or adjusting for smoking effects in studies of other risk factors that often have more subtle effects, having an objective biomarker to aid in classification of smoking status is useful.

A biomarker of sustained smoking during pregnancy will also be useful in studies of childhood health outcomes where DNA can be obtained from routinely collected neonatal blood spots. Concomitant information on smoking in birth certificates or medical charts is often limited to yes or no during pregnancy and may have large numbers of missing values. Smoking during pregnancy queried several years later when children have had time to develop conditions that are known to be related to parental smoking is subject to biased reporting.

We previously reported that sustained maternal smoking during pregnancy has a much greater effect on newborn methylation than smoking that ceased early in pregnancy (Joubert et al. 2014). Here we show that sustained smoking during pregnancy had a greater effect on birth weight than any smoking during pregnancy, which was not significantly related to birth weight. The smoking methylation score we developed, which reflects sustained rather than any smoking, may better capture health effects of maternal smoking on the newborn as our birth weight analysis suggests.

Given the large and reproducible impact of maternal smoking on the newborn methylome, there is great interest in whether these signals mediate health outcomes causally linked to this exposure, such as reduced birth weight (Küpers et al. 2015). However, regardless of whether they are mediators, these methylation signals are useful biomarkers of *in utero* exposure. The success of this approach for smoking, where methylation signals are abundant, augurs well for the use of the methylation data to develop objective biomarkers of *in utero* exposures that are harder to measure and may have subtler effects on the epigenome and child health outcomes.

We note that the smoking methylation score was developed using data from a homogenous population from Norway. Therefore, we do not know how generalizable it would be to other ethnic groups. However, the training and test methylation data sets were generated at different time points in different analytic batches spaced about 2 years apart. Thus our finding of good performance of the score in the test set incorporates the effects of laboratory variability increasing the applicability to other studies.

To develop the score, we used data that were not normalized (not corrected for the fact that the Illumina®450K includes two probe types). We did this both for comparability with our previous publication (Joubert et al. 2012) and to increase generalizability to studies that may not have normalized or used varying normalization procedures. We found that normalizing using the popular β-mixture quantile normalization (BMIQ) method (Teschendorff et al. 2013) does not influence the smoking results in our data (Joubert et al. 2014). In addition, Wu et al. (2014), using our data, found that when examining an association with a high level of statistical significance, such as maternal smoking in pregnancy, results using raw versus normalized data are very similar. In addition, we did not batch correct the test and training sets which were analyzed at different points in time. We did this to better approximate how the score will behave in other studies to increase generalizability of our results. For investigators who might want to normalize to our data, we provide the mean methylation values for the set of CpGs used in the score in Table S9.

As a supplemental analysis, we performed the LASSO method using the log ratios, rather than the untransformed methylation beta values, and the model performance was virtually identical (training: untransformed accuracy = 0.96 vs. log ratio accuracy = 0.95; test: untransformed accuracy = 0.91 vs. log ratio accuracy = 0.91; see Table S6); however, it retained more CpGs (37 vs. 28). A score with fewer elements is easier to use, but for users who prefer to analyze their data on the log ratio scale, we provide a supplementary table with the 37 CpGs and their coefficients (see Table S10).

We refer to our primary exposure metric, based on the combination of a positive self report and cotinine measured in samples taken at approximately 18 weeks, as sustained smoking because most women who reported that they had smoked in early pregnancy but quit later, had done so by 18 weeks. However, to determine sustained smoking, it would have been better to have measured cotinine again near the end of pregnancy.

A limitation in developing a methylation score biomarker of sustained smoking during pregnancy is that there is no clear gold standard. Cotinine is only a reliable biomarker of recent smoking. We primarily used cotinine to train the model (since only a few cotinine-based nonsmokers were switched to smokers based on self-report) and thus our score cannot perform better than cotinine. This removes our ability to discern whether the methylation score is truly superior to cotinine, a short-term biomarker, in predicting health effects of sustained maternal smoking on birth weight or other outcomes.

## Conclusions

We have developed a novel biomarker in the newborn of exposure to sustained maternal smoking during pregnancy using Illumina®450K DNA methylation data. This methylation score is an objective biomarker that reflects much longer-term exposure than cotinine, the best available smoking biomarker. The score can be easily implemented in other studies with similar methylation data. It provides a means to validate self-reported nonsmoking status during pregnancy and enables the ascertainment of sustained smoking when limited time course information was collected. This biomarker of sustained smoking during pregnancy should facilitate better adjustment for maternal smoking in studies of other *in utero* exposures with more subtle effects and may improve the ability to capture novel health effects caused by this important prenatal exposure.

## Supplemental Material

(283 KB) PDF
