# Supplementary material for: DNA Methylation Score as a Biomarker in Newborns for Sustained Maternal Smoking during Pregnancy
Source: Environ Health Perspect. 2016 Jun 21;125(4):760–6. doi: 10.1289/EHP333 (PMC5381987; doi:10.1289/EHP333)
Supplement: (283 KB) PDF [file EHP333.s001.acco.pdf]

Environ Health Perspect
DOI: 10.1289/EHP333

Note to readers with disabilities: *EHP* strives to ensure that all journal content is accessible to all readers. However, some figures and Supplemental Material published in *EHP* articles may not conform to 508 standards due to the complexity of the information being presented. If you need assistance accessing journal content, please contact ehp508@niehs.nih.gov. Our staff will work with you to assess and meet your accessibility needs within 3 working days.

Supplemental Material

DNA Methylation Score as a Biomarker in Newborns for Sustained Maternal Smoking during Pregnancy

Sarah E. Reese, Shanshan Zhao, Michael C. Wu, Bonnie R. Joubert, Christine L. Parr, Siri E. Håberg, Per Magne Ueland, Roy M. Nilsen, Øivind Midttun, Stein Emil Vollset, Shyamal D. Peddada, Wenche Nystad, and Stephanie J. London

Table of Contents

Figure S1: Density distribution of the smoking methylation score for the training set (A; N=1,057) and test set (B; N=221). Black solid line - all individuals; Blue dashed line – combined sustained smoking non-smokers; Red dotted line - combined sustained smoking smokers; Grey vertical dashed line indicates the score threshold.

Figure S2: Plot showing the combined sustained smoking model ROC curves for the training (red solid line) and test (black dotted line) datasets. Cutoff score of -0.37 developed in the training set is shown on both curves, along with the corresponding specificity and sensitivity.

Table S1: 28 CpGs selected by the logistic LASSO regression model using combined sustained smoking.

Table S2: Cross-tabulation of cotinine-based and self-reported sustained smoking

Table S3: 24 CpGs selected by the logistic LASSO regression model using cotinine-based sustained smoking.

Table S4: 12 CpGs selected by the logistic LASSO regression model using self-reported sustained smoking.

Table S5: 26 CpGs selected by the logistic LASSO regression model using combined sustained smoking restricted to CpGs that overlap with the IlluminaEPIC BeadChip.

Table S6: Logistic LASSO results for main model (combined sustained smoking), the main model using log ratios, and the main model using only the CpGs available on the IlluminaEPIC array– The number of CpGs (q) used to calculate the smoking methylation score, area under the curve (AUC) and 95% confidence interval (CI), smoking methylation score threshold, percent accuracy and CI, sensitivity and CI, specificity and CI, and the number and percentage of false negatives (FN) and false positives (FP).

Table S7: Descriptive statistics of smoking variables in the training data set.

Table S8: Descriptive statistics of birth weight and maternal and newborn characteristics in the training data set.

Table S9: Mean Methylation Values of the 28 CpGs selected by the main model (combined sustained smoking).

Table S10: 37 CpGs selected by the logistic LASSO regression model on the log ratios using combined sustained smoking.

Supplementary Figures

Figure S1: Density distribution of the smoking methylation score for the training set (A; N=1,057) and test set (B; N=221). Black solid line - all individuals; Blue dashed line – combined sustained smoking non-smokers; Red dotted line - combined sustained smoking smokers; Grey vertical dashed line indicates the score threshold.

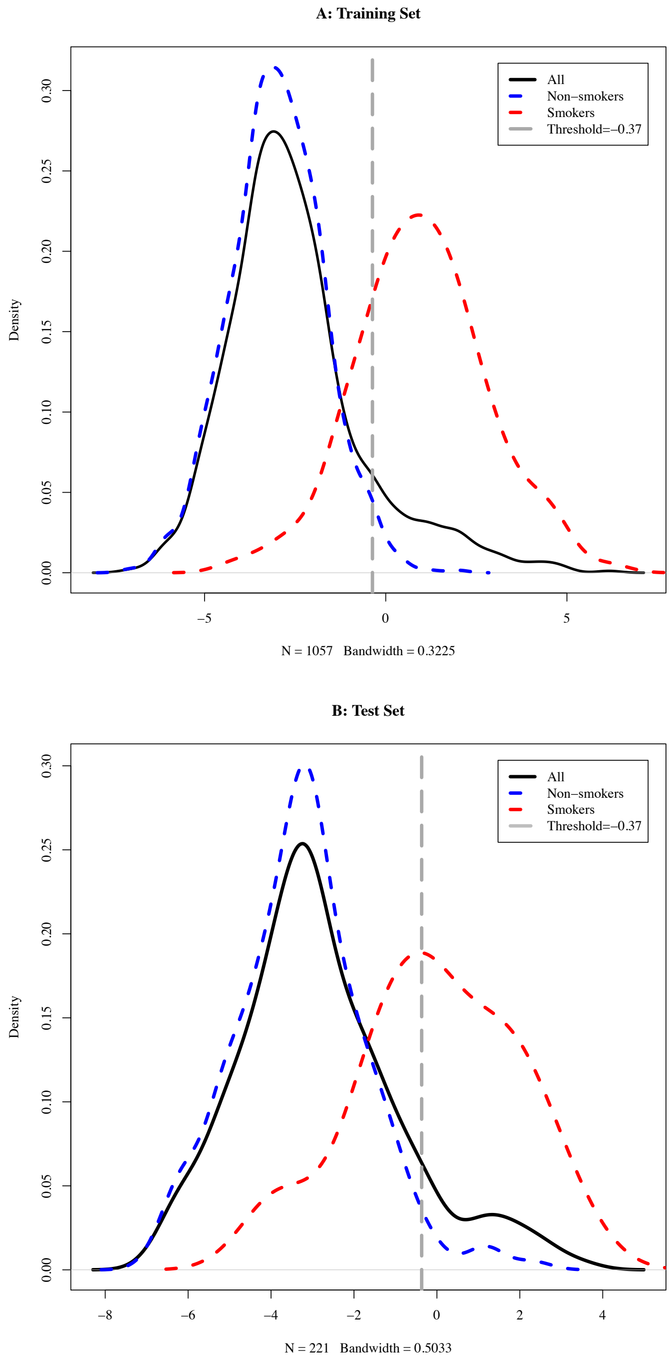

Figure S2: Plot showing the combined sustained smoking model ROC curves for the training (red solid line) and test (black dotted line) datasets. Cutoff score of -0.37 developed in the training set is shown on both curves, along with the corresponding specificity and sensitivity.

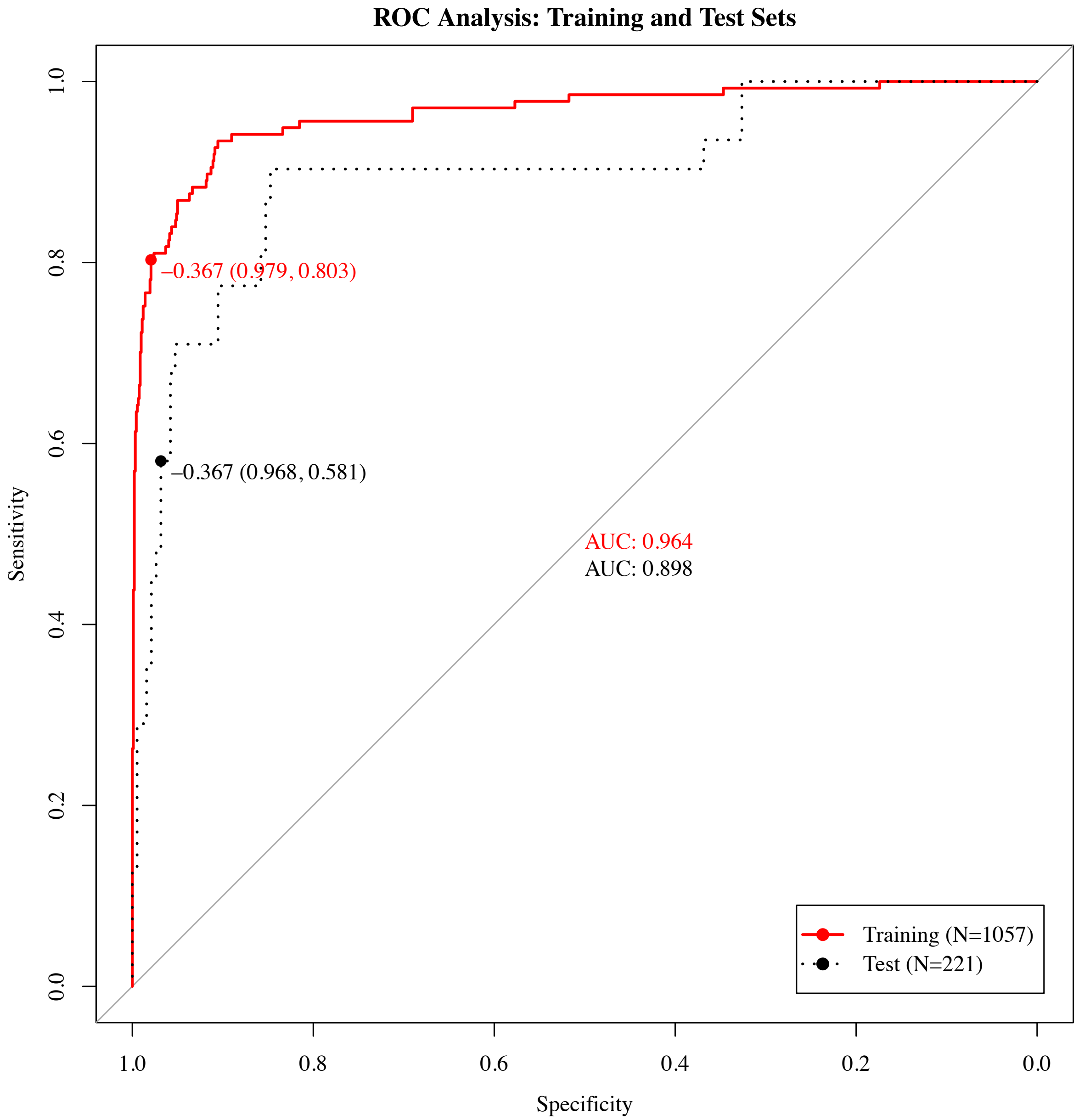

Supplementary Tables

Table S1: 28 CpGs selected by the logistic LASSO regression model using combined sustained smoking*.

CpG	Coefficient	Gene Name
**cg00709966**	-1.667	
**cg02256631**	-0.191	*ITGAM*
**cg02482603**	2.706	*RABGAP1L*
**cg04103532**	1.786	*HIVEP2*
**cg04180046**	14.027	*MYO1G*
**cg04506190**	2.318	*PLXND1*
**cg05549655**	6.210	*CYP1A1*
**cg05575921**	-10.909	*AHRR*
**cg08698721**	1.142	*MEG3*
**cg09743950**	-6.330	*ITGAM*
**cg10799846**	-4.963	*SYNJ2*
**cg11864574**	-0.370	*SPAG6*
**cg12186702**	3.847	*PLVAP*
**cg13834112**	1.514	
**cg13893782**	-0.963	*PDE7B*
**cg14179389**	-6.304	*GFI1*
**cg14351425**	6.361	*GRK5*
**cg14633298**	5.050	*TRIM27*
**cg14743346**	2.286	*DERL1*
**cg17397069**	-2.912	*SGCD*
**cg19381766**	5.245	
**cg22154659**	-0.773	*HOXA1*
**cg22802102**	-0.254	*NTF3*
**cg23304605**	0.011	*CCDC88C*
**cg25189904**	-3.903	*GNG12*
**cg25949550**	-46.991	*CNTNAP2*
**cg26764244**	-0.246	*GNG12*
**cg27291468**	0.836	

* In the combined sustained smoking variable, a woman's positive report of daily smoking during pregnancy overrides a cotinine value of ≤56.8 nmol/L used to classify a woman as a non-smoker in the cotinine-based sustained smoking variable.

Table S2: Cross-tabulation of cotinine-based and self-reported sustained smoking

Cotinine-based Sustained Smoking	Self-reported Sustained Smoking	Training (MoBa1; n (%))	Test (MoBa2; n (%))
Non-smoker	Non-smoker	910 (86.1)	186 (84.2)
Non-smoker	Smoker	20 (1.9)	5 (2.3)
Smoker	Non-smoker	26 (2.5)	5 (2.3)
Smoker	Smoker	101 (9.5)	25 (11.3)

Table S3: 24 CpGs selected by the logistic LASSO regression model using cotinine-based sustained smoking.

**CpG**	**Coefficient**	**Gene Name**
**cg00044354**	-0.657	*MIER1;WDR78*
**cg00709966**	-1.145	
**cg04180046**	12.144	*MYO1G*
**cg04506190**	2.072	*PLXND1*
**cg05371791**	-2.670	*ANKMY1*
**cg05549655**	5.353	*CYP1A1*
**cg05575921**	-12.766	*AHRR*
**cg06686709**	6.381	*CACNB4*
**cg07339236**	-0.052	*ATP9A*
**cg08872493**	-0.139	*HTR1D*
**cg09743950**	-3.092	*ITGAM*
**cg09935388**	-3.588	*GFI1*
**cg12803068**	0.122	*MYO1G*
**cg12806681**	-0.464	*AHRR*
**cg13834112**	2.253	
**cg14179389**	-6.339	*GFI1*
**cg14351425**	5.049	*GRK5*
**cg19381766**	4.924	
**cg20294319**	3.181	*LRCH1*
**cg22154659**	-2.932	*HOXA1*
**cg22875872**	4.801	*PTPRF*
**cg25189904**	-2.030	*GNG12*
**cg25949550**	-38.434	*CNTNAP2*
**cg26764244**	-1.471	*GNG12*

Table S4: 12 CpGs selected by the logistic LASSO regression model using self-reported sustained smoking

**CpG**	**Coefficient**	**Gene Name**
**cg00253658**	1.361	
**cg04180046**	8.403	*MYO1G*
**cg05549655**	3.467	*CYP1A1*
**cg05575921**	-11.139	*AHRR*
**cg09743950**	-1.710	*ITGAM*
**cg09935388**	-2.675	*GFI1*
**cg11429111**	0.125	
**cg13787193**	-13.339	*LOC148709*
**cg14179389**	-1.518	*GFI1*
**cg17681313**	-4.307	*CYP20A1*
**cg23304605**	0.681	*CCDC88C*
**cg25949550**	-38.772	*CNTNAP2*

Table S5: 26 CpGs selected by the logistic LASSO regression model using combined sustained smoking restricted to CpGs that overlap with the IlluminaEPIC BeadChip.

**CpG**	**Coefficient**	**Gene Name**
**cg02256631**	-0.191	*ITGAM*
**cg02482603**	2.706	*RABGAP1L*
**cg04103532**	1.786	*HIVEP2*
**cg04180046**	14.027	*MYO1G*
**cg04506190**	2.318	*PLXND1*
**cg05549655**	6.210	*CYP1A1*
**cg05575921**	-10.909	*AHRR*
**cg08698721**	1.142	*MEG3*
**cg09743950**	-6.330	*ITGAM*
**cg10799846**	-4.963	*SYNJ2*
**cg12186702**	3.847	*PLVAP*
**cg13834112**	1.514	
**cg13893782**	-0.963	*PDE7B*
**cg14179389**	-6.304	*GFI1*
**cg14351425**	6.361	*GRK5*
**cg14633298**	5.050	*TRIM27*
**cg14743346**	2.286	*DERL1*
**cg17397069**	-2.912	*SGCD*
**cg19381766**	5.245	
**cg22154659**	-0.773	*HOXA1*
**cg22802102**	-0.254	*NTF3*
**cg23304605**	0.011	*CCDC88C*
**cg25189904**	-3.903	*GNG12*
**cg25949550**	-46.991	*CNTNAP2*
**cg26764244**	-0.246	*GNG12*
**cg27291468**	0.836	

Table S6: Logistic LASSO results for main model (combined sustained smoking), the main model using log ratios, and the main model using only the CpGs available on the IlluminaEPIC array– The number of CpGs (q) used to calculate the smoking methylation score, area under the curve (AUC) and 95% confidence interval (CI), smoking methylation score threshold, percent accuracy and CI, sensitivity and CI, specificity and CI, and the number and percentage of false negatives (FN) and false positives (FP).

**Model**		***q***	**AUC (CI)**	**Threshold**	**Accuracy (CI)**	**Sensitivity (CI)**	**Specificity (CI)**	**FN** (%)	**FP** (%)
**Combined Sustained Smoking**	Training	28	0.96 (0.95,0.98)	-0.37	0.96 (0.94,0.97)	0.80 (0.74,0.87)	0.98 (0.97,0.99)	27 (2.6)	19 (1.8)
	Test		0.90 (0.83,0.97)		0.91 (0.88,0.95)	0.58 (0.39,0.74)	0.97 (0.94,0.99)	13 (5.9)	6 (2.7)
**Combined Sustained Smoking – log ratios**	Training	37	0.97 (0.95,0.99)	7.41	0.95 (0.94,0.96)	0.82 (0.76,0.88)	0.97 (0.96,0.98)	24 (2.2)	27 (2.6)
	Test		0.90 (0.82,0.97)		0.91 (0.88,0.95)	0.58 (0.39,0.74)	0.97 (0.94,0.99)	13 (5.9)	6 (2.7)
**Combined Sustained Smoking – EPIC**	Training	26	0.96 (0.95,0.98)	0.33	0.95 (0.94,0.96)	0.81 (0.75,0.88)	0.97 (0.96,0.98)	26 (2.5)	27 (2.6)
	Test		0.90 (0.83,0.97)		0.91 (0.88,0.95)	0.65 (0.45,0.81)	0.96 (0.93,0.98)	11 (5.0)	8 (3.6)

Table S7: Descriptive statistics of smoking variables in the training data set*.

**Variable**		**Training**
**Methylation Score Class (n (%))**	No	907 (87.3)
	Yes	132 (12.7)
**Cotinine-based Sustained Smoking (n (%))**	No	912 (87.8)
	Yes	127 (12.2)
**Self-reported Sustained Smoking (n (%))**	No	918 (88.4)
	Yes	121 (11.6)
**Combined Sustained Smoking (n (%))**	No	902 (86.8)
	Yes	137 (13.2)
**Self-reported Any Smoking (n (%))**	No	745 (71.7)
	Yes	294 (28.3)

* Note: Participants with nonmissing data for all covariates were used for the birth weight analyses; therefore, the sample counts in this table are slightly different for the smoking variables between the main and sensitivity analyses (N=1,057; Table 1) compared with the birth weight analyses (N=1,039).

Table S8: Descriptive statistics of birth weight and maternal and newborn characteristics in the training data set.

Variable	Training
**N***	1039
**Birth Weight (g) (mean ± sd)**	3644 ± 543.6
**Gestational Age (weeks) (mean ± sd)**	40.0 ± 1.6
**Mother's Age (years) (mean ± sd)**	29.9 ± 4.3
**Sex - Male (n (%))**	550 (52.9)
**Parity ≥ 1 (n (%))**	617 (59.4)
**Maternal Education**	
**<High School (n (%))**	78 (7.5)
**High School (n (%))**	334 (32.1)
**Some College (n (%))**	460 (44.3)
**+4yr College (n (%))**	167 (16.1)

* Note: Participants with nonmissing data for all covariates were used for the birth weight analyses; therefore, the total sample size was slightly lower for the birth weight analyses (N=1,039).

Table S9: Mean Methylation Values of the 28 CpGs selected by the main model (combined sustained smoking).

CpG	Mean
**cg00709966**	0.4105
**cg02256631**	0.1028
**cg02482603**	0.4274
**cg04103532**	0.5712
**cg04180046**	0.4513
**cg04506190**	0.2577
**cg05549655**	0.1966
**cg05575921**	0.8649
**cg08698721**	0.6021
**cg09743950**	0.6816
**cg10799846**	0.2261
**cg11864574**	0.4560
**cg12186702**	0.5048
**cg13834112**	0.5733
**cg13893782**	0.1812
**cg14179389**	0.2388
**cg14351425**	0.2197
**cg14633298**	0.8567
**cg14743346**	0.7659
**cg17397069**	0.6746
**cg19381766**	0.5556
**cg22154659**	0.4234
**cg22802102**	0.7330
**cg23304605**	0.1827
**cg25189904**	0.5317
**cg25949550**	0.1111
**cg26764244**	0.2733
**cg27291468**	0.6715

Table S10: 37 CpGs selected by the logistic LASSO regression model on the log ratios using combined sustained smoking.

CpG	Coefficient	Gene Name
**cg00044354**	-0.036	*MIER1;WDR78*
**cg00709966**	-0.476	
**cg02256631**	-0.108	*ITGAM*
**cg02482603**	0.906	*RABGAP1L*
**cg02973307**	-0.015	*KCTD15*
**cg03991871**	-0.639	*AHRR*
**cg04103532**	0.518	*HIVEP2*
**cg04180046**	3.000	*MYO1G*
**cg04506190**	0.596	*PLXND1*
**cg05549655**	1.022	*CYP1A1*
**cg05575921**	-1.328	*AHRR*
**cg05757757**	0.103	
**cg05854217**	-0.093	
**cg08698721**	0.234	*MEG3*
**cg09273054**	-0.021	*MAL2*
**cg09743950**	-1.508	*ITGAM*
**cg10673740**	0.180	*BAG3*
**cg10799846**	-0.969	*SYNJ2*
**cg11348106**	0.085	*SEC14L1*
**cg11864574**	-0.283	*SPAG6*
**cg12186702**	1.254	*PLVAP*
**cg13834112**	0.846	
**cg13893782**	-0.164	*PDE7B*
**cg14179389**	-1.220	*GFI1*
**cg14351425**	1.214	*GRK5*
**cg14633298**	0.855	*TRIM27*
**cg14743346**	0.339	*DERL1*
**cg17397069**	-0.706	*SGCD*
**cg18422587**	0.187	
**cg19381766**	1.214	
**cg22154659**	-0.277	*HOXA1*
**cg23304605**	0.125	*CCDC88C*
**cg25189904**	-1.113	*GNG12*
**cg25949550**	-4.500	*CNTNAP2*
**cg26038465**	0.111	*MAFF*
**cg26709433**	0.012	
**cg27291468**	0.195	
